# Recent Progress of Wearable Triboelectric Nanogenerator-Based Sensor for Pulse Wave Monitoring

**DOI:** 10.3390/s24010036

**Published:** 2023-12-20

**Authors:** Yiming Wang, Xiaoke Wang, Shijin Nie, Keyu Meng, Zhiming Lin

**Affiliations:** 1 School of Electronic and Information Engineering, Southwest University, Chongqing 400715, China; wym0608@email.swu.edu.cn (Y.W.); wangxk@email.swu.edu.cn (X.W.); nsjnsj@email.swu.edu.cn (S.N.); 2 School of Electronic and Information Engineering, Changchun University, Changchun 130022, China; mengky@ccu.edu.cn

**Keywords:** triboelectric nanogenerator, self-powered sensor, cardiovascular diseases, pulse wave

## Abstract

Today, cardiovascular diseases threaten human health worldwide. In clinical practice, it has been concluded that analyzing the pulse waveform can provide clinically valuable information for the diagnosis of cardiovascular diseases. Accordingly, continuous and accurate monitoring of the pulse wave is essential for the prevention and detection of cardiovascular diseases. Wearable triboelectric nanogenerators (TENGs) are emerging as a pulse wave monitoring biotechnology due to their compelling characteristics, including being self-powered, light-weight, and wear-resistant, as well as featuring user-friendliness and superior sensitivity. Herein, a comprehensive review is conducted on the progress of wearable TENGs for pulse wave monitoring. Firstly, the four modes of operation of TENG are briefly described. Secondly, TENGs for pulse wave monitoring are classified into two categories, namely wearable flexible film-based TENG sensors and textile-based TENG sensors. Next, the materials, fabrication methods, working mechanisms, and experimental performance of various TENG-based sensors are summarized. It concludes by comparing the characteristics of the two types of TENGs and discussing the potential development and challenges of TENG-based sensors in the diagnosis of cardiovascular diseases and personalized healthcare.

## 1. Introduction

The World Health Organization estimates that approximately 17.9 million people die of cardiovascular diseases each year, accounting for 31% of global deaths (WHO is the directing and coordinating agency on international health issues within the United Nations system). It is obvious that cardiovascular diseases are one of the most urgent threats to human health. Today, cardiovascular diseases (CVDs) have become the leading cause of death in the world, including coronary heart disease (CHD), heart failure (HF), stroke, hypertension, and arrhythmia. It is estimated that 17.9 million people die from CVDs every year, and the data from the World Heart Federation (WHF) show that more than 500 million people suffer from CVDs worldwide [1,2]. Generally, the risk of developing cardiovascular disease increases with age, and it is more common in people over the age of 40. With an increasingly aging global population, it is foreseeable that deaths from CVDs will continue to increase [3,4]. In addition, the world has a large population with risk factors such as obesity, smoking, lack of exercise, and dyslipidemia [5]. It is undeniable that all of these risk factors have some potential to cause cardiovascular diseases. It is also worth noting that CVDs are not limited to the elderly but increasingly affect young people. Unhealthy lifestyles and excessive physical activity contribute to the increased incidence of CVDs in young people [6]. However, patients show symptoms that are not obvious before most outbreaks of cardiovascular diseases. Minor discomforts are often overlooked, which are the cause of delays in optimal treatment. It is one of the reasons for the high mortality associated with cardiovascular disease. Therefore, the majority of cardiovascular diseases can be prevented through early detection. In order to save patients with CVDs in time, it is essential to develop wearable sensors for pulse wave monitoring to diagnose CVDs. Wearable triboelectric nanogenerators designed for pulse wave monitoring offer a potential solution to this problem, which can be worn by individuals conveniently, enabling continuous monitoring of pulse waves. They will detect abnormalities in patients and alert the patient and healthcare providers in times of danger [7].

Analyzing pulse waveforms can provide clinically valuable information for the diagnosis of cardiovascular diseases, including heart rate (HR), pulse wave velocity (PWV), and blood pressure (BP). There are various parameters that play important roles in the diagnosis of cardiovascular diseases, including arrhythmia, coronary artery disease, and arterial hypertension [8,9]. The process of monitoring cardiovascular diseases is simplified by analyzing the pulse wave to obtain relevant and valuable information. Currently, 24 h Holter monitoring is used for long-term cardiovascular monitoring in clinical practice, which requires multiple electrodes to be attached to the patient’s chest and connected to the recording system through wires [10]; however, 24 h Holter monitoring suffers from high cost, complex operation, and bulkiness [11]. Physicians frequently employ cuff-based blood pressure monitors, which can only measure blood pressure intermittently [12]. Electrocardiography (ECG) [13] and Photo Plethysmo Graphy (PPG) [14] are the two primary methods used by the majority of heart rate sensors [15]. Although PPG is a noninvasive test that uses photoelectricity to detect changing blood volume in living tissue, it can yield incorrect measurements due to factors such as variations in the color of the skin, the distance from the artery, and interference from ambient light [16,17]. Traditional methods of diagnosing cardiovascular diseases have some limitations and are not suitable for long-term continuous heart rate monitoring. In order to continuously and noninvasively monitor pulse waves without interfering with daily activities, flexible and lightweight wearable sensors are required [18,19].

**Figure 1:**
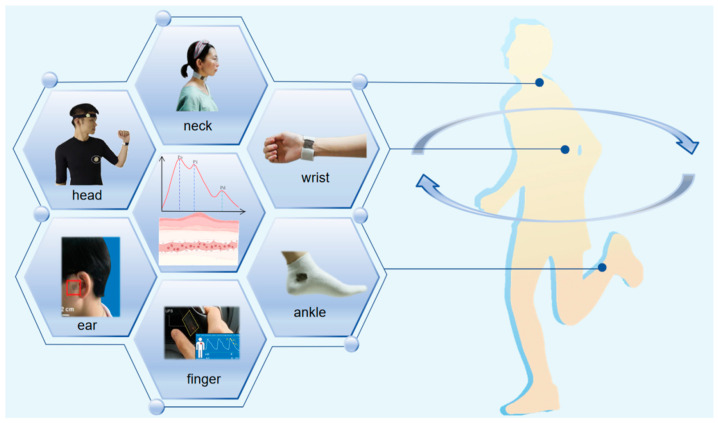
Wearable TENGs for pulse wave monitoring can be placed on different human body parts, such as the head, neck, behind the ear, wrists, fingertips, and ankles.

During human movement, some wearable pulse wave sensors might be affected by motion artifacts due to inadequate adhesion and a lack of a conformal interface with human skin. As a result, the accuracy of measurement results will be affected. TENGs for pulse wave monitoring can accommodate the high degree of deformation and twisting that occurs in human arterial vessels under high static forces, and the effects of motion artifacts are reduced by structural improvements, including structures such as kirigami, hemispherical arrays, pyramidal arrays, and nanowires [20,21,22,23,24]. Especially, TENG-based pulse wave monitoring sensors improve the disadvantages of traditional electronic devices and have the following advantages over traditional monitoring devices: self-powered, long service life, superior sensitivity, and comfortability. Additionally, scientists are currently working on researching the wearable TENG for pulse wave monitoring that can measure pulse waves accurately, noninvasively, and over the long term [25,26]. Herein, as illustrated in Figure 1, the evolution of TENG-based sensors for pulse wave monitoring sensors over the past few years is conducted. Firstly, four modes of operation of TENG will be elaborated. Secondly, in this investigation, TENGs for pulse wave monitoring are classified into two categories, namely wearable flexible film-based TENG sensors and textile-based TENGs for pulse wave monitoring. Thirdly, the characteristics of the various TENG-based pulse wave monitoring sensors are described in detail, including the preparation material, the manufacturing method, the working mechanism, the experimental performance obtained, and synthetically compared their advantages. Finally, some of the current shortcomings and directions for improvement of TENG-based pulse wave monitoring are summarized and presented. The future directions of TENG-based pulse wave monitoring sensors are discussed from five aspects: comfortability, sensitivity, stability, communication and internet, and large-scale fabrication with reasonable cost. Moreover, this review concludes that TENGs will become a key solution for human health monitoring and provide more convenient medical devices for CVD diagnosis and therapy.

## 2. Wearable TENG for Pulse Wave Monitoring

### 2.1. Mechanism of TENGs

**Figure 2:**
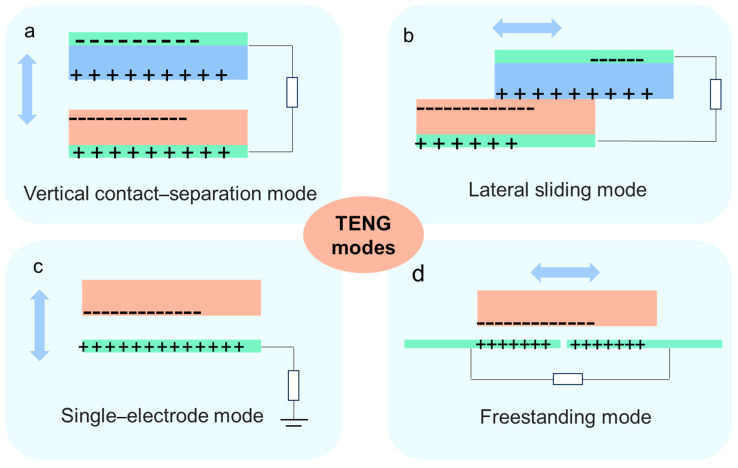
Four modes of operation and device structure of TENG. (**a**) Vertical contact–separation mode. (**b**) Lateral sliding mode. (**c**) Single–electrode mode. (**d**) Freestanding mode.

After the proposal of Wang’s team in 2012, TENG has been growing rapidly [27,28]. TENGs are used to convert external mechanical energy into electricity through the triboelectric effect and electrostatic induction [29,30,31,32,33]. The triboelectric effect requires the use of two materials with different electron affinities. The power generation process at TENG could be simply described as the transfer of electrons between two different materials in frictional contact, and the aggregation of electrons into a charged layer, which results in potential difference and the formation of an electric current [34,35]. The mechanism of TENG is divided into four basic working modes [36], including vertical contact–separation mode [37], lateral sliding mode [38], single-electrode mode [39], and freestanding mode [40]. As depicted in Figure 2a, the vertical contact–separation mode works by repeating contact and separating between two oppositely charged materials. As shown in Figure 2b, the lateral sliding mode uses the sliding of two materials to create displacement to form a flow of electrons, which results in the formation of an alternating current. As illustrated in Figure 2b, the single-electrode mode does not require the connection of two materials; only one end is required, which can be used as an electrode, and an existing grounded end as a negative electrode to generate the current. As shown in Figure 2d, freestanding mode requires the use of a symmetrical pair of electrodes, and alternating currents are generated between the two electrodes due to electrostatic induction. Currently, the most commonly used modes for TENG are the vertical contact–separation and the single-electrode mode [41].

Because of its unique operating principle and significant advantages, including high efficiency, low cost, lightweight, and environmental friendliness, it is widely used in various fields, including wearable electronics, energy harvesting, and self-powered sensing [42,43,44,45]. Due to the differences in TENG materials, structures, and practical applications, TENGs for pulse wave monitoring are classified into two categories in this investigation, namely wearable flexible film-based TENGs and textile-based TENGs for pulse wave monitoring sensors. Next, following the development process of TENG over the last few years, we described each TENG for pulse wave monitoring in detail, including the preparation materials, the manufacturing process, the working mechanism, the experimentally obtained performance, and the distinct characteristics of each sensor.

### 2.2. Flexible Film-Based Wearable TENG for Pulse Wave Monitoring

**Figure 3:**
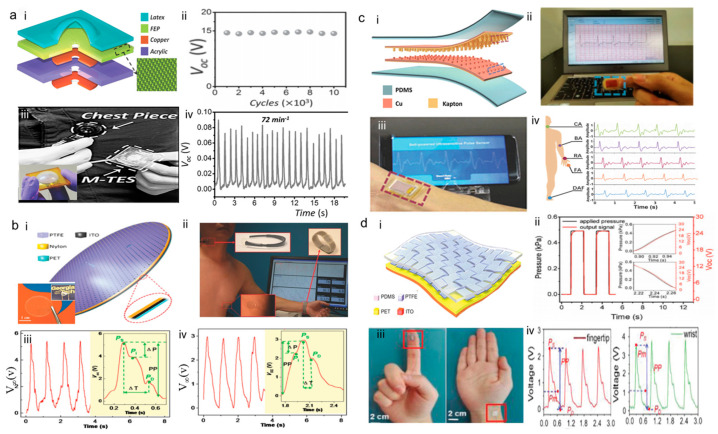
Flexible film-based wearable TENG for pulse wave monitoring. (**a**) Structure, sensitivity, and experimental measurement results of pulse wave at a site for M-TES as pulse wave monitor. (i) Schematic of the M-TES. (ii) Stability of the M-TES after longtime operation. (iii) Setup of the M-TES as a heartbeat monitor. (iv) Output voltage signals of the M-TES as a heartbeat monitor. Reproduced with permission from ref. [46]. (**b**) Structure, sensitivity, and experimental measurement results of pulse wave at a site for BMS as pulse wave monitor. (i) Schematic of the BMS. (ii) A photograph showing the bionic membrane sensors was directly attached to simultaneously monitor the pulse waves of the participant from his carotid artery, chest, and wrist. (iii) The real-time voltage outputs when the sensors are placed over the carotid arteries of a 30-year-old man. (iv) The real-time voltage outputs when the sensors are placed over the carotid arteries of a 70-year-old man. Reproduced with permission from ref. [47]. (**c**) Structure, sensitivity, and experimental measurement of pulse wave results at a site for SUPS as pulse wave monitor. (i) Schematic of the SUPS. (ii) The real-time signal outputs when SUPS are placed over the finger artery. (iii) The real-time signal outputs when SUPS are placed over the wrist artery. (iv) The signal output of SUPS pressed on various artery position. Reproduced with permission from ref. [48]. (**d**) Structure, sensitivity, and experimental pulse wave results of WCSPS as pulse wave monitor at a site. (i) Schematic of the WCSPS. (ii) WCSPS response time characterization. (iii) WCSPS is worn directly on the fingertip and wrist to measure human pulse wave signals. (iv) The real-time signal outputs when WCSPS are placed over the finger and wrist, respectively. Reproduced with permission from ref. [49].

In 2014, a membrane-based triboelectric sensor (M-TES) for surveillance and healthcare monitoring was researched [46]. It was the first wearable TENG for pulse wave monitoring with a multi-layer structure consisting of fluorinated ethylene propylene (FEP), a copper layer, and a latex membrane. M-TES relies on changes in air pressure within the membrane to sense frictional charge. To study the stability of M-TES, researchers conducted multiple cycles of tests, and after 10,000 cycles, the monitoring results remained relatively stable. The M-TES is more sensitive when its size is smaller, having an average sensitivity of 0.04 mV Pa^−1^. As shown in Figure 3a, the M-TES was worn on the chest of an adult male, and the measured heart rate was 72 beats per minute (bpm). It is accurate and effective when compared to traditional heart rate monitoring devices. This suggests that M-TES could be used in pulse wave monitoring and personal healthcare.

In 2015, Yang et al. [47] introduced a self-powered bionic membrane sensor (BMS). It was inspired by the structure of the human eardrum, coupling the contact electrified cation effect with a structure inspired by a human eardrum. BMS is a multi-layer thin-film elliptical structure, and the working mode is a single-electrode mode. It consists of an indium tin oxide (ITO) electrode, a nylon-charged layer supported by a thin layer of polyethylene terephthalate (PET), and a polytetrafluoroethylene (PTFE) layer. In addition, the researchers performed several repeated cycles and found that the signal of the BMS changed almost negligibly after 40,000 cycles, as demonstrated in Figure 3b; thus, BMS demonstrated stability and durability. BMS holds a superior sensitivity of 51 mV Pa^−1^ with a fast response time of less than 6 ms, as well as a pressure detection limit down to 2.5 Pa. To test the feasibility of the BMS for long-term health monitoring, a thirty-year-old adult male was monitored for ten minutes with a wrist pulse wave, as depicted in Figure 3b, during the measurement process, the tester’s measurement results were accurate. Accordingly, BMS opens up a new dimension in the field of wearable medical health monitoring.

In 2017, the flexible self-powered ultrasensitive pulse sensor (SUPS) was researched, SUPS can be used in non-invasive multi-indicator cardiovascular monitoring [48]. To effectively address the issues of sweat immersion and electrostatic interference, the as-fabricated SUPS consisted of two friction layers, electrode layers, and a spacer, and all device structures were encapsulated inside by elastomer. SUPS has excellent output performance (1.52 V), high peak signal-to-noise ratio (45 dB), long-term performance (107 cycles), and low cost. As shown in Figure 3c, the experimenter performed real-time wrist and fingertip pulse wave measurements and obtained accurate results. It has been experimentally shown that SUPS has the advantage of measuring PWV, a property that makes SUPS promising for use in the diagnosis of CVDs such as atherosclerosis, arrhythmia, and arteriosclerosis.

In 2018, Meng et al. [49] reported a flexible weaving constructed self-power pressure sensor (WCSPS) that can be used to capture subtle mechanical changes in blood pressure in blood vessels and represent them as electrical signals of human pulse waves. The as-fabricated WCSPS consists of multi-layer nanostructures etched and cross-programmed by nanowire plasma. WCSPS enhanced surface roughness by etching nanowires and enhanced electrical output signals to sense more subtle human changes in blood pressure. The high stability of the WCSPS, as observed in 40,000 cycles, allowed for long-term monitoring. For enhancing human comfortableness and portability, the WCSPS could be worn on fingers and ankles to capture weaker bio-signals. As depicted in Figure 3d, WCSPS holds an ultra-sensitivity of 45.7 mV Pa^−1^ with an ultrafast response time of less than 5 ms. The high stability and sensitivity of the WCSPS has been demonstrated to allow long-term monitoring of pulse wave.

**Figure 4:**
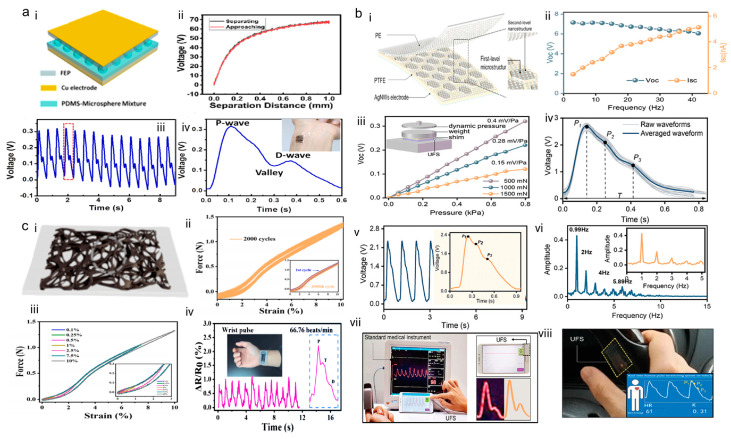
Flexible film-based wearable TENG for pulse wave monitoring. (**a**) Structure, sensitivity, and pulse wave experimental measurements of microsphere-expanded TENG used as pulse wave monitoring. (i) Schematic of the microsphere-expanded TENG. (ii) Change of output voltage during the separating and approaching processes of the PDMS-microsphere layer and the FEP film in one cycle. (iii) Voltage signals recorded during the pulse rate monitoring test. (iv) The real-time signal outputs when microsphere-expanded TENG are placed over the wrist. Reproduced with permission from ref. [50]. (**b**) Structure, sensitivity, and pulse wave experimental measurements of UFS used as in-vehicle pulse wave monitoring. (i) Schematic of the UFS. (ii) Voltage and current changes in the UFS under an applied pressure with frequencies from 1–40 Hz. (iii) The capacity of the UFS in response to dynamic pressures under static forces (500, 1000, and 1500 mN). (iv) The acquired fingertip pulse waves from each day over 30 days. (v) The details of the fingertip pulse (containing three peak points) captured by the UFS. (vi) The frequency spectrum of a 28-year-old man’s fingertip pulse waveform. (vii) Measurement of the fingertip pulse using the UFS. (viii) Demonstration of fingertip pulse waveform monitoring on the surface of the car steering wheel. Reproduced with permission from ref. [51]. (**c**) Structure, sensitivity, and experimental pulse wave measurements of green and cost-effective carbonized lucerne (CL) used for pulse wave monitoring. (i) Schematic of the CL. (ii) Stability of the CL after longtime operation. (iii) Force-strain curves of the encapsulated CL film under various strains at a stretching speed of 5 mm min^−1^. (iv) The real-time signal outputs when CL are placed over the wrist. Reproduced with permission from ref. [52].

In 2019, Hu et al. [50] invented a triboelectric nanogenerator based on swellable microspheres in a polydimethylsiloxane (PDMS) mixture for pulse wave monitoring. The structure is characterized by the incorporation of a micro-structure of expandable microspheres, which enhances the sensitivity of the sensor. It works by adjusting the weight percentage of expandable microspheres in the PDMS to obtain different sensitivities. The experimental results show that the higher the mixing ratio, the higher the sensitivity. The maximum sensitivity of the sensor can reach 150 mV Pa^−1^. In the article, a test experiment was conducted in which the tester monitored the pulse wave of the radial artery at the wrist, as shown in Figure 4a. The sensor could be able to respond to subtle pressure deformations in the human body to obtain an accurate pulse waveform. In general, the sensor shows excellent sensitivity and stability compared to the previous TENG, and the fabrication process is simpler and cost-effective. Accordingly, the sensor demonstrates great potential and value in the timely monitoring and diagnosis of CVDs.

In 2021, an ultra-thin and flexible sensor (UFS) with the features of high flexibility was introduced [51], featuring shape-adaptability, ultra-broad range, and high-pressure sensitivity for unconstrained precision pulse wave sensing. The researcher summarized the strengths and weaknesses of commercially available film-based wearable TENGs. The as-fabricated UFS is a single-electrode mode and a double-layer nanostructure. It was experimentally verified that the sensitivity of UFS was as high as 0.15 mV Pa^−1^ under the dynamics of 0~0.8 kPa, and the fingertip briefly touching the stay could be measured by UFS to receive the valid pulse wave. As a flexible sensor for finger-touching unconstrained biomonitoring, the sensing performance of UFS is compromised by its bendability; to test the performance of the UFS, 2000 folding, twisting, and spraying tests were carried out. The UFS maintains excellent sensing performance under these extreme conditions, as illustrated in Figure 4b. The results after 30 consecutive days of measurements on subjects are quite stable, confirming the possibility, accuracy, and applicability of the UFS for long-term monitoring of human fingertip pulse waves. It has been demonstrated that due to the high flexibility and shape adaptability of the UFS, an in-vehicle healthcare system based on the UFS and fully integrated bio-health management with mobile phones is possible.

In 2022, a film structure for a carbonized loofah (CL) with flexible strain sensors was developed to monitor pulse waves [52]. The process of preparing the CL film can be briefly described as follows: firstly, 2 cm × 3 cm slices of loofah need to be cut from the dried loofah sponge; subsequently, the loofah is carbonized through a three-step carbonization process for electrical conductivity; finally, it is placed in acrylic acid for encapsulation and curing. The as-fabricated CL film can be used as strain sensors and single-electrode TENGs. Therefore, the CL strain sensor exhibits advanced characteristics, such as ultra-high sensitivity. As demonstrated in Figure 3b, the average response time is 30 ms, indicating that it has good sensitivity, and it has an ultra-low detection limit (0.01% strain). After 2000 cycle tests, it was also concluded that it has good durability. The study also demonstrated that due to the high flexibility and shape adaptability of the sensor, loofah branding-based pulse wave monitoring sensors can be worn on the human body for long periods while accurately obtaining biological information.

**Figure 5:**
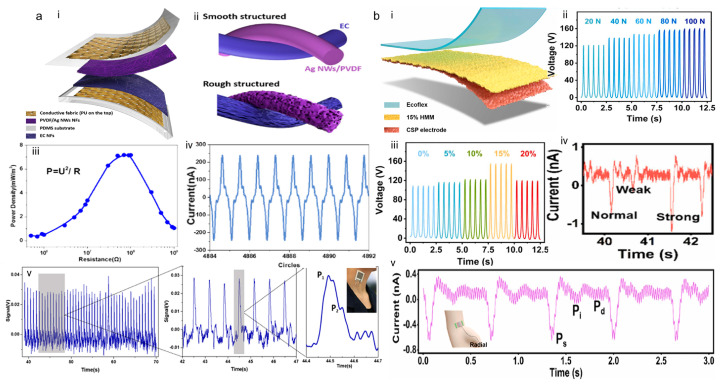
Flexible film-based wearable TENG for pulse wave monitoring. (**a**) Structure, sensitivity, and pulse wave experimental measurements of a triboelectric all-fiber structured pressure sensor used as pulse wave monitoring. (i) Schematic of the sensor textile. (ii) Comparison of the morphology difference of the triboelectric nanofibers with the smooth and rough surface. (iii) Instantaneous power output of the sensor textile on external load resistances. (iv) Stability of the sensor textile after longtime operation. (v) The real-time signal outputs when the sensor textile are placed over the wrist. Reproduced with permission from ref. [56]. (**b**) Structure, sensitivity, and pulse wave experimental measurements of SUVSs used as in-vehicle pulse wave monitoring. (i) Schematic of the SUVSs. (ii) Open circuit voltage working in different contact force. (iii) Open circuit voltage of H-S-TENGs with different HPC contents. (iv) The zoomed-in three waves of carotid artery from an arrhythmia patient. (v) Pulse signal of radial artery from a male volunteer detecting by SUVSs. Reproduced with permission from ref. [57].

Electrospun nanofibers have the advantages of excellent uniformity, large porosity, and large surface area, which provide a new choice for the triboelectric layer of TENG and can significantly improve the performance and wearability of TENGs. Therefore, wearable TENG-based monitoring pulse wave sensors fabricated by simple electrostatic spinning technology will become a highly desirable strategy for CVD diagnosis and therapy [53,54,55]. In 2020, Ding et al. fabricated a triboelectric all-fiber structured pressure sensor via a facile electrospinning technique [56]. The sensor textile holds a composite structure made up of a polyvinylidene fluoride/Ag nanowire nanofibrous membrane (NFM), an ethyl cellulose NFM, and two layers of conductive fabrics. The constructed sensor operated in vertical contact–separation mode, and introduced a hierarchically rough structure on the nano-fibers to give the sensor high shape adaptability. As shown in Figure 5a, the sensor has a sensitivity of up to 1.67 and 0.2 mV Pa^−1^ in the pressure ranges of 0–3 and 3–32 kPa and shows excellent mechanical stability after 7200 cycles of continuous operation. Experimenters placed the sensor on the neck of a healthy 35-year-old woman and measured clear and distinct pulse waveforms. Accordingly, the fabric pressure sensor provides an excellent means of long-term monitoring and treatment of cardiovascular disease.

In 2022, Dai et al. reported a facile and controllable water-assisted electrospinning approach to fabricate hierarchical morphology mats (HMMs) for self-powered sensor applications [57]. A single-electrode TENG (H-S-TENG) was then assembled using the HMMs. The fabricated H-S-TENG prepared by 15% HMMs exhibits a high output power density of 3.37 W/m^2^ and excellent durability during more than 10,000 contact–separation cycles. Based on H-S-TENG, the developed self-powered ultrasensitive vibration sensor (SUVS) achieves accurate detection of pulse waves from different arteries. As shown in Figure 5b, the SUVSs tested a female volunteer and obtained the 60 s carotid pulse wave; tested a male volunteer and obtained carotid, brachial, and radial artery pulse waves; and monitored a patient with cardiac arrhythmia. The 60 s irregular pulse wave was obtained in the radial artery. The experimental results appeared to be accurate, demonstrating the excellent performance of SUVSs in pulse detection.

Flexible film-based wearable TENGs for pulse wave monitoring are capable of continuous, accurate, and real-time monitoring of pulse wave signals from the human body. Because of their portability and comfort, they can be securely attached to human skin. The various flexible film-based wearable TENGs for pulse wave monitoring described above have been tested in several experiments on diverse test populations of different ages. According to our summary findings, flexible film-based wearable TENGs for pulse wave monitoring exhibited superior sensitivity and durability. Scientists believe that as TENGs continue to develop, they will gradually become important devices in the diagnosis and prevention of cardiovascular disease.

### 2.3. Textile-Based Wearable TENG for Pulse Wave Monitoring

Due to cardiovascular disease happening suddenly and delaying medical treatment, it is a major threat to human health. Therefore, the long-term and real-time monitoring of pulse waves of specific populations (including the elderly, athletes, and patients with underlying pathologies) is essential [58,59]. Currently, wearable self-powered flexible film-based TENGs for pulse wave monitoring are lightweight and inexpensive. However, they may be affected by factors such as human sweat and movement, which can be a source of inaccuracy and discomfort over long periods of wear [60]. Therefore, researchers have improved and designed wearable self-powered textile TENGs for pulse wave monitoring to address these drawbacks. Textile TENGs can be integrated into clothing, bed linen, quilts, and other household items, where they demonstrate the advantages of being softer, more comfortable, and more sustainable. These compelling characteristics make textile TENGs a superior human health monitoring device for accurate, self-powered, bio-compatible, and cost-effective pulse wave monitoring with wearing comfort superior to traditional wearable electronics [61]. In this review, textile-based TENGs are systematically investigated and introduced as an emerging biotechnology for the application of wearable pulse wave monitoring.

**Figure 6:**
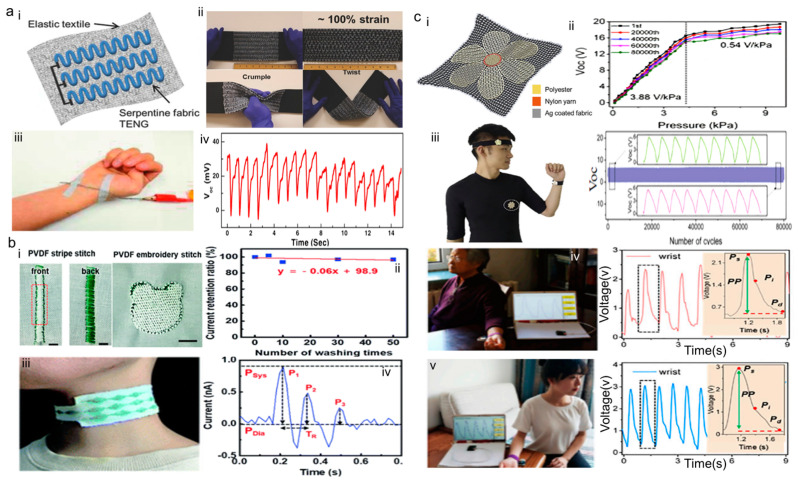
Textile-based wearable TENG for pulse wave monitoring. (**a**) Structure, sensitivity, and experimental pulse wave measurements of SEHT used as pulse wave monitoring. (i) Schematic of the SEHT. The upper left arrow points to the elastic textile, and the lower right arrow points to the serpentine fabric TENG. (ii) Photographs of device with demonstrations of being different mechanical forces, including folding, twisting, and crumpling. (iii) SEHT are placed over the wrist. (iv) The real-time signal outputs when the SEHT are placed over the wrist. Reproduced with permission from ref. [62]. (**b**) Structure, sensitivity, and experimental pulse wave mentoring of textile TENG produced on sewing machines. (i) Schematic of the textile TENG produced on sewing machines. Sutured traces are shown in the dotted box. (ii) Stability of the sensor textile after longtime operation. (iii) The sensor is placed over the neck. (iv) The real-time signal outputs when the sensor textile is placed over the neck. Reproduced with permission from ref. [63]. (**c**) Structure, sensitivity, and experimental pulse wave measurements of TS. (i) Schematic of the TS. (ii) Stability of the TS after longtime operation. (iii) The TS are placed over the head, chest, wrist. (iv) The real-time voltage outputs when the TS are placed over the wrist arteries of a 75-year-old woman. (v) The real-time voltage outputs when the TS are placed over the wrist arteries of a 22-year-old woman. Reproduced with permission from ref. [64].

In 2017, a new type of ‘’single’’ thread-based triboelectric nanogenerator was developed. This is an elastic, large-area, and highly stretchable energy-harvesting textile (SEHT) [62]. SEHT is a textile made from rust-resistant, wash-resistant, stable materials by bending and weaving. The working mechanism of SEHT is a single-electrode mode, and SEHT increases the area of contact with the body and facilitates energy harvesting. Experimenters tested the stability of SEHT through multiple folding, twisting, creasing, and washing machine cleaning tests. As shown in Figure 6a, the SEHT was not damaged and still showed flexibility, high tensile strength, and robustness. The as-fabricated SEHT can accurately monitor pulse waves, which is the first time that the threaded triboelectric nanogenerator sensor has been successfully used for healthcare monitoring. Researchers suggested that SEHT could be sewn onto clothing cuffs and used in the medical field as a wrist pulse monitor, which can be worn for long-term pulse wave monitoring.

In 2018, Hyun-Hyuk Ko [63] published a textile-based wearable TENG with the advantage of being produced in high volumes by sewing machines and in a wide variety of pattern colors. After the many tests carried out, the resulting textile-based TENG showed superior mechanical stability after being tested for folding, stretching, twisting, and creasing deformation, as well as machine washability. It is worth proposing that the TENG could be placed on various parts of the human body for pulse wave monitoring. As shown in Figure 6b, after several experiments, the measurements were shown to be consistent with the pulse wave detected by traditional methods. It has the potential to be used as a self-powered wearable electronic to monitor pulse waves. This TENG paved the way for lightweight, low-cost, and large-scale manufacturing of self-powered wearable TENGs for pulse wave monitoring in commercial clothing and garments.

In 2020, a textile-based sensor (TS) was presented that has a stable bilayer structure constructed in the form of a flower [64]. The design of the special flower shape improves the TS’s ability to sense pulse waves and increases the overall sensitivity. The TS can be directly sewn onto clothing for enhanced aesthetics, and measures pulse waves on the forehead, wrist, and chest. As demonstrated in Figure 6c, the as-fabricated TS has demonstrated an excellent sensitivity of 3.88 V/kPa and has proven to have mechanical stability and water resistance in pressure and immersion tests. TS continuously catches very small pulse waves, symbolizing the wide range of possibilities for wearable textile TENGs in the diagnosis of cardiovascular disease in the elderly and disabled.

**Figure 7:**
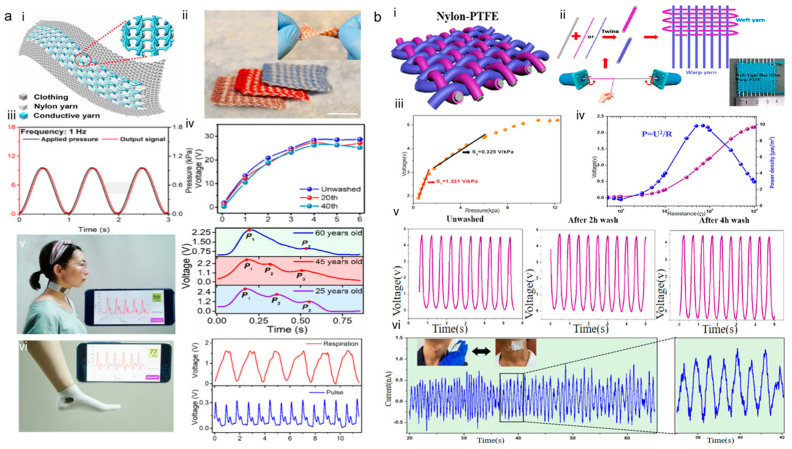
Textile-based wearable TENG for pulse wave monitoring. (**a**) Structure, sensitivity, and experimental pulse wave measurements of TATSA. (i) Schematic of the TATSA. (ii) Photograph of TATSAs in different colors (scale bar, 2 cm). The inset is the twisted TATSA. (iii) Plots showing the frequency responses under a dynamic pressure of 1 kPa and pressure input frequency of 1 Hz. (iv) Output characteristics of the TATSA after washing 20 and 40 times. (v) The real-time voltage outputs when the TATSA are placed over the wrist arteries of a woman. (vi) The real-time voltage outputs when the TATSA are placed over the ankle arteries. Reproduced with permission from ref. [65]. (**b**) Structure, sensitivity, and experimental pulse wave measurements of self-powered friction-electric sensing textile. (i) Schematic of the self-powered friction-electric sensing textile. (ii) Schematic of the fabrication process of the core-shell yarns. (iii) Output voltage as a function of the applied pressure. (iv) Output voltage and instantaneous power output of the sensing textile on different external load resistances. (v) A comparison of output voltage for the sensing textile after 2 h and up to 4 h washing. (vi) Real-time detection of the pulse wave and magnified pulse wave of the carotid artery using the sensing textile. Reproduced with permission from ref. [66].

Textile-based TENGs can also include TENGs where the entire textile is a TENG; for example, the triboelectric all-textile sensor array (TATSA) with high-pressure sensitivity and comfort for monitoring pulse waves [65]. TATSA works in the contact–separation mode and takes a special geometric weave to increase the contact area between the textile and the body for enhancing conductivity. Its conductive yarn and nylon material are stronger and more resistant to stretching, and the sensor can be mass-produced. As depicted in Figure 7a, TATSA has been subjected to numerous twist, pull, and folding tests without the properties being affected. TATSA was proven to have excellent flexibility and tensile properties. The repeatability, stability, and washability of the TATSA were proven by 100,000 cycles. The sensitivity of the TATSA was 7.84 mV Pa^−1^ and the response time was less than 20 ms. The TATSA was used to detect pulse waves in different age groups, which can be used for further analysis of cardiovascular parameters to assess the cardiovascular health of the test subjects. TATSA represents a step forward in the development of wearable textile electronics.

In 2020, Li et al. [66] developed a triboelectric sensing textile constructed with core–shell yarns with a twisted spiral structure that facilitates the withstanding of various deformations. The most important feature of the sensor is the complete fiber construction without the use of any adhesives or closed metal membranes; it has excellent breathability and is comfortable compared to previous wearable textile sensors. As illustrated in Figure 7b, the sensing textile has a sensitivity of 1.33V·kPa^−1^ and 0.32V·kPa^−1^ in the pressure range of 1.95–3.13 kPa and 3.20–4.61 kPa. Respectively, it was proven to have better mechanical stability and sensing capability even after 4200 cycles and 4 h of continuous washing. The breathable, washable, and dye-able sensing textile can be easily attached to the neck to record physiological signals of the arterial pulse wave to reflect current human health conditions. This research provided an innovative and promising track for multifunctional textile monitoring healthcare sensors that could be used in the timely monitoring of pulse waves and the diagnosis of CVDs.

In the aforementioned report, the textile-based TENG sensors for wearable pulse wave monitoring demonstrate superior sensitivity and user-friendliness. The textile-based TENGs can be integrated into everyday products including clothing, and they demonstrate the advantages of being softer, more comfortable, and breathable. In the development of textile-based TENGs, they have demonstrated stability and durability through various tests, such as machine washing, stretching, and creasing. If the research is successful and effective in gradually improving the performance of textile-based TENGs, it may enhance the applicability of future textile-based sensors to reliably and accurately monitor the pulse wave signals of the human body. Furthermore, these measurements may even be reliable under extreme conditions.

## 3. Conclusions and Future Perspectives

### 3.1. Conclusions

Cardiovascular diseases are the leading cause of death in the world, and arterial pulse wave is an important diagnostic tool for assessing CVDs. Continuous and accurate monitoring of pulse waves is essential for the prevention and detection of CVDs [67,68]. The shortcomings of traditional monitoring electronics have led to the realization that they are no longer sufficient to meet people’s daily demands [69]. Therefore, TENG improved on the shortcomings of traditional electronics for monitoring pulse waves, such as being battery-powered, bulky, having a short lifespan, and instability in long-term monitoring. With the development of TENGs, the diversity of materials has led to a significant increase in sensitivity and breathability, and the monitoring results obtained have become more accurate [70,71,72]. This review provides an in-depth summary of relevant self-powered wearable TENGs for real-time pulse wave monitoring and cardiovascular disease diagnosis. The performances of the presented TENG-based pulse wave monitoring sensors are listed in Table 1.

In this investigation, TENGs for pulse wave monitoring are classified into two categories, namely wearable flexible film-based TENGs and textile-based TENGs for pulse wave monitoring sensors. Subsequently, TENGs for pulse wave monitoring are described in terms of their preparation, manufacturing method, working principle, experimental performance, and specific characteristics. In summary, the benefits of TENG for pulse wave monitoring include having a long service life, superior sensitivity, and excellent comfortability, as well as being self-powered. It has the ability to accurately detect human pulse wave signals and facilitate the subsequent analysis of pulse waves to obtain clinically useful information, such as PWV, HR, and BP. Polymer films or fabrics are commonly used as substrates for wearable TENG sensors, which greatly improve the user-friendliness and breathability of the sensors and offer the possibility of long-term CVD diagnosis and therapy. After summarizing the development of TENGs in recent years, we believe that TENG-based medical electronic devices will have great potential to replace the traditional devices for monitoring pulse waves. Therefore, we also believe that there is progress and potential for the development of TENGs in the field of healthcare. To sum up, TENGs have certain advantages in the long-term monitoring of pulse waves for diagnosis of CVDs, and TENGs will potentially provide a new means for the future of intelligent medicine.

### 3.2. Future Perspectives

Wearable TENGs for pulse wave monitoring are a more convenient and accurate method of long-term monitoring for cardiovascular disease [73,74,75]. Despite being studied thoroughly over the past few years in the academic community, TENGs for pulse wave monitoring face significant challenges in clinical practice. Wearable TENGs for pulse wave monitoring should be improved according to the tester’s feelings and demands after being worn by the tester [76]. For instance, comfort and aesthetics are the foremost concerns when it comes to wearing TENGs for extended periods, followed by accuracy, stability, and connectivity with technology [77]. Next, we look at the future of TENG for pulse wave monitoring, which proposes solutions to the major challenges facing TENG before clinical application, making TENG more suitable for long-term human wear and daily use in a wide range of applications.

#### 3.2.1. Comfortability

Due to the clinical characteristics of CVD (long-term and sudden onset), there are current demands for TENGs that can be worn in the long term and continuously monitor pulse waves. Today, we find that flexible film-based TENG lacks breathability and aesthetics, which is not conducive to long-term wearing, and textile-based TENG lacks stability and softness. Therefore, the future of wearable TENGs should focus more on comfortability [78,79]. We can improve the breathability, softness, and stretchability by changing the materials and microstructure of the flexible film-based TENG to avoid the patient feeling uncomfortable or affecting their daily activities [80,81]. In the future, it will be possible to reduce its weight and increase its elasticity and softness by changing the weave structures and materials of the textile-based TENG. Starting with the shaping and coloring of the textile-based TENG, it will be possible to improve the aesthetics [82,83].

#### 3.2.2. Sensitivity

When monitoring pulse waveforms, the monitoring results will be affected by the people themselves, leading to inaccurate measurements. For example, when measuring the elderly population, the pulse waves cannot be accurately captured due to their loose skin. In addition, small fluctuations in arterial pressure are not easily detected in people with different levels of body fat. As a result, the tightness of the textile-based TENG can be adjusted to fit the human body, promoting sensitivity and accurately converting pulse waves into electrical signals [84,85]. In the future, researchers can increase the sensitivity of TENG by changing its structure and material. In the structure, microstructures or nanostructures can be designed, including wrinkles, hemisphere arrays, pyramid arrays, and nanowires, which can increase the surface charge and promote intimate contact with the opposite material [86,87,88]. In materials, the ability to dope, inject, and coat with extrinsic chemicals can be used to adjust and optimize the surface charge affinity and ultimately achieve higher output voltage [89,90]. From a textile TENG perspective, the weave structure of the textile can be altered to increase sensitivity [91], adjusting the weave density of the textile-based TENG to better match the curvature of the human body’s muscles. This is one way to increase sensitivity and accurately convert the pulse wave into an electrical signal.

#### 3.2.3. Stability

In people’s daily activities, textile-based TENGs are prone to generating periodic mechanical changes with human movement, while flexible film-based TENGs are prone to bending and breaking [92,93]. In addition, TENGs can be affected by factors such as sweat erosion and temperature changes, which can decrease the accuracy of monitoring pulse waves and potentially reduce their lifespan [94]. Currently, most textile-based TENGs cannot be washed or are damaged by washing. To enhance their long-term stability, we could add a layer of waterproof encapsulant made of special materials to reduce the impact of the external environment on the TENG [95,96,97,98].

#### 3.2.4. Communications and Internet

Today, in order to improve the ability of the TENG for pulse wave monitoring to acquire and store important clinical information, the future development of wearable TENGs for pulse wave monitoring may be linked to the Internet of Things (IoT) [99,100,101]. The transmission, integration, and storage of data through the IoT will be more conducive to the formation of a cloud database in healthcare. When TENGs are combined with IoT to form a cloud healthcare information system, the real-time pulse wave data by TENGs can be transmitted to mobile devices, such as smartphones and notebooks, and stored in an internet cloud information repository. Hence, wherever they are in the world, medical professionals can receive timely pulse wave data to determine the wearer’s physical condition [102,103]. The system can compare daily pulse information and track its changes to alert doctors and patients when abnormalities occur.

Currently, wearable TENGs for pulse wave monitoring show the potential for the diagnosis of cardiovascular diseases [104], but the potential of the devices is questionable in clinical practice because they still do not allow doctors to save the life of the user who is in danger [105]. When TENGs are combined with artificial intelligence (AI) to create an AI medical assistant, they can monitor pulse waves and analyze abnormalities. If a user is in danger, the medical assistant could determine the user’s condition to provide basic self-help treatments and contact the nearest hospital to provide valuable time for subsequent treatment by a doctor.

#### 3.2.5. Large-Scale Fabrication with Reasonable Cost

TENG-based pulse wave sensors are being studied intensively, with the ultimate goal of commercialization and medical application. The research process should also take into account the cost of mass production. High relative humidity poses a barrier to the commercialization of TENGs. Effectively improving the hydrophobicity of TENGs, such as by selecting materials with superior waterproofing properties, could be a crucial step in advancing TENGs toward commercialization. The friction charge density of TENGs depends on the contact between the friction surfaces (the closer the contact, the higher the friction charge), and the wear between the surfaces will reduce the durability of the TENGs. If the durability of the TENG is improved, the device’s lifespan will be extended, which will facilitate its commercialization. Researchers and scientists have found that wear can be reduced and durability can be improved through various methods, such as liquid lubrication, electrostatic rolling, and centrifugal force [106]. On the other hand, a suitable packaging design is necessary to ensure the performance of TENGs in severe environments. A suitable packaging design will increase the lifespan of the sensor and facilitate its mass commercialization more effectively [107]. The complete encapsulation of the TENG improves its waterproofing and washability, making it more suitable for everyday use. What is more, with the development of TENGs, the large-scale commercialization of TENGs is one step closer. Scientists also expect that in the near future, TENGs will provide a more convenient method for helping to treat and diagnose CVDs [108,109,110].

To sum up, the use of TENGs for pulse wave monitoring allows for the accurate detection of human pulse wave signals. Their ability to accurately capture pulse waves provides ease of subsequent pulse wave analysis and smooth acquisition of clinically useful information, such as PWV, HR, and BP. Polymer films or fabrics are commonly used as substrates for wearable TENG sensors, which greatly improve the user-friendliness and breathability of the sensors and offer the possibility of long-term CVD diagnosis and therapy. When TENGs are combined with artificial intelligence (AI), the system can apply algorithms to build CVD databases that record a person’s clinical information, including heart rate, pulse rate, and blood pressure. The aforementioned AI medical assistant can use this data to determine the user’s current physical condition, alerting them in time to avoid delaying medical attention. Combining TENGs for pulse wave monitoring with IoT and AI, which is the inevitable trend in the future of the technological society, not only improves the efficiency of doctors’ rescuing patients but also promotes social harmony. It is clear that the trends in academic research and marketing practice herald a huge leap forward in the field of CVD diagnosis. Wearable TENGs for pulse wave monitoring are an essential part of the development of IoT in the healthcare industry and have a wide development scope and commercial value.

## Figures and Tables

**Table 1:** The performances of TENG-based pulse wave monitoring sensors are listed.

Sensor	Voltage (V)	Current (μ A)	Sensitivity or Efficiency (mV Pa^−1^)	Response Time (ms)	Ref.
M-TES	14.5		0.04		[46]
BMS	2	0.02	51		[47]
SUPS	109		0.55		[48]
WCSPS	15		45.7	5	[49]
TENG based on swellable microspheres in a polydimethylsiloxane mixture for pulse wave monitoring	70		150		[50]
UFS	47	35	0.7	4	[51]
CL	20	10		<30	[52]
Triboelectric all-fiber structured pressure sensor	25	255	1.67	5	[56]
SUVSs	3.82	130	3.37	<30	[57]
SEHT	200	200	7.5	10	[62]
PVDF stitch-based triboelectric textile sensors	9.8	140			[63]
TS	18		3.88	5	[64]
TATSA	30	100	7.84	<20	[65]
Triboelectric sensing textile constructed with core-shell yarns	5	26	0.32	5	[66]

## Data Availability

Data are contained within the article.
